# Glycogen Phosphorylase: A Drug Target of Amino Alcohols in *Echinococcus granulosus*, Predicted by a Computer-Aided Method

**DOI:** 10.3389/fmicb.2020.557039

**Published:** 2020-11-23

**Authors:** Congshan Liu, Jianhai Yin, Wei Hu, Haobing Zhang

**Affiliations:** ^1^Key Laboratory of Parasite and Vector Biology, National Institute of Parasitic Diseases, Chinese Center for Disease Control and Prevention, Ministry of Health (MOH), National Center for International Research on Tropical Diseases, World Health Organization (WHO) Collaborating Centre for Tropical Diseases, Shanghai, China; ^2^Department of Microbiology and Microbial Engineering, School of Life Sciences, Fudan University, Shanghai, China

**Keywords:** echinococcosis, amino alcohols, drug target, inverse docking, glycogen phosphorylase

## Abstract

Echinococcosis is an important parasitic disease that threats human health and animal husbandry worldwide. However, the low cure rate of clinical drugs for this disease is a challenge. Hence, novel compounds and specific drug targets are urgently needed. In this study, we identified drug targets of amino alcohols with effects on *Echinococcus* species. The drug targets were predicted with the idTarget web server. Corresponding three-dimensional structures of the drug targets were built after sequence BLAST analysis and homology modeling. After further screening by molecular docking, the activities of the candidate targets were validated *in vitro*. We ultimately identified glycogen phosphorylase as a potential drug target for amino alcohols. There are two genes coding glycogen phosphorylase in *Echinococcus granulosus* (EgGp1 and EgGp2). EgGp1 was abundant in *E. granulosus* PSCs, while EgGp2 was abundant in the cysts. These proteins were located at suckers and somas of *E. granulosus* PSCs and near the rostellum of cysts developed from PSCs. The effective compounds docked into a pocket consisting of E124, K543 and K654 and affected (either inhibited or enhanced) the activity of *E. granulosus* glycogen phosphorylase. In this study, we designed a method to predict drug targets for echinococcosis treatment based on inverse docking. The candidate targets found by this method can contribute not only to understanding of the modes of action of amino alcohols but also to modeling-aided drug design based on targets.

## Introduction

Echinococcosis, which is caused by larval-stage tapeworms in the genus *Echinococcus*, severely affects human health and animal husbandry worldwide. Two of the main *Echinococcus* spp., *Echinococcus granulosus* and *Echinococcus multilocularis*, cause cystic echinococcosis (CE) and alveolar echinococcosis (AE), respectively. Echinococcosis is a neglected tropical zoonotic disease, but the disability adjusted life year (DALY) was estimated to be 871,000 in 2010 (Budke et al., 2017). Humans acquire this disease via consumption of egg-contaminated food and water. The growth and metastasis of the parasites cause lesions in organs and can even cause death in severe cases. However, the therapeutic agents for this disease are limited to only two clinically used drugs, albendazole, and mebendazole, both of which have poor cure rates. Hence, novel compounds and specific drug targets are urgently needed.

Currently, the discovery of novel drugs for echinococcosis is still mostly dependent on whole-organism screening. In this method, the activities of compounds are tested on protoscoleces (PSCs) or metacestodes *in vitro*, and the effects are then validated in a parasite-infected mouse model. The disadvantage of this phenotypic screening method is its laborious and inefficient nature. The target-based drug screening (Croston, 2017), which is a faster, easier, and less costly than whole-organism screening, could potentially enhance echinococcosis drug research. However, identification of promising drug targets is important for target-based drug screening.

Two different strategies are part of the ongoing effort to advance drug target identification. The first and most widely used strategy is confirmation of the important functions of macromolecules. The commonly used techniques include homology analysis (Hung and Weng, 2016; Flo et al., 2017), molecular docking (Esteves and Paulino, 2013; Vadloori et al., 2018), differential gene expression analysis (DeMarco and Verjovski-Almeida, 2009; Parkinson et al., 2012; Saurabh et al., 2014), and RNA interference (RNAi; Misra et al., 2017; Mousavi et al., 2019), among other techniques. The second strategy involves drug target identification based on active compounds. In this strategy, inverse molecular docking is used to predict potential drug targets according to the structures of known compounds in protein databases. The interactions of small molecules and bio-macromolecules are evaluated by their binding energies and docking conformations (Kharkar et al., 2014; Lee et al., 2016). Several drug targets have been identified by this method, such as retinoic acid receptor alpha and GTPase HRas, which are targets for Danshen (Chen, 2014; Chen and Ren, 2014); FabG, FabI and FabZ in fatty acid biosynthesis pathways, which are targets for the antimalarial drug flavone and its derivatives (Kumar et al., 2014); and matrix metalloproteinase-9 and other tumor-related proteins, which are targets for marine compounds (Chen et al., 2017). Possible drug targets can also be found by comparing differences in gene expression levels after drug treatment. For example, the expression of P-glycoprotein in *Cooperia oncophora* is increased under ivermectin treatment, and doxycycline treatment inhibits the expression of mitochondrial and plastid proteins in *Plasmodium falciparum*, suggesting that these proteins can be further studied as drug targets (Briolant et al., 2010; Areskog et al., 2013).

With the increasing understanding of the biology and genetics of *Echinococcus* species, several drug targets have been predicted, such as tubulin, which is considered to selectively bind to benzimidazoles (Lacey, 1990). In addition, the proteasome of *E. multilocularis* metacestodes has been found to be inhibited by bortezomib *in vitro* (Stadelmann et al., 2014), and various proteins that play crucial roles in signaling pathways (Gelmedin et al., 2008; Epping and Brehm, 2011) and electron transport and antioxidant systems (Salinas et al., 2017; Wang et al., 2018) are considered to be promising drug targets of *E. granulosus* and *E. multilocularis*. In our previous study, we reported a series of amino alcohols with potential effects on *Echinococcus* spp. (Liu et al., 2018; Liu et al., 2020). To predict the drug targets of these compounds, idTarget, a web server for inverse docking, was used in the current study. The potential targets were further identified by a BLAST search against the echinococcal genome, homology modeling and molecular docking and were preliminaryly validated with related molecular biological experiments.

## Materials and Methods

### Compounds and Reagents

The amino alcohol compound (JF16) was purchased from Enamine, Ltd. (Kievska Region, Ukraine) with purity > 90%. Mefloquine (MEF, Sigma, United States), ursolic acid (UA, Sigma) and other reagents (unless specifically stated) were obtained from Sigma (United States).

### Protein Target Prediction by idTarget and Criteria for Candidate Target Selection

idTarget is freely available at http://idtarget.rcas.sinica.edu.tw/ (Wang et al., 2012). The files for 11 effective amino alcohols (Supplementary Figure 1A) were submitted to the idTarget server. The fast mode was used for the protein set, and AM1-BCC/AmberPAR M99SB was used as the ligand/protein charge model. The other parameters were set as the default values.

The results downloaded from idTarget included all of information for docking of the compounds with proteins in the protein data bank (PDB). The inverse docking result list presents only the top 200 proteins based on their binding energy. To identify the common drug targets of the amino alcohols, we set the following criteria for proteins selection: (1) the protein ranked within the first 200 results and appeared more than six times among the results for the 11 effective compounds; (2) the protein ranked among the first 100 proteins in the inverse docking results for MEF and JF16 (two of the most effective amino alcohols); and (3) the binding energy was < -9 kcal/mol. All the three-dimensional (3D) structures and sequences of the proteins were downloaded from the PDB^1^.

### Identification of the Potential Drug Targets by Homology Modeling and Molecular Docking

#### BLAST Search With the *E. granulosus* Genome and Homology Modeling

*Echinococcus granulosus* genome data were downloaded from the Sanger Institute website^2^. Then, sequence alignment was carried out with BLAST 2.2.25^3^. A sequence with bit score > 100 was considered homologous. Then, the sequence alignments were used to generate homology models with MODELLER 9.13^4^, and SWISS-MODEL^5^. After optimization with Chimera 1.9, the homology models were evaluated using the Structural Analysis and Verification Server SAVES^6^.

#### Molecular Docking

The interactions and docking poses of the candidate proteins with MEF and JF16 were determined with AutoDock 4.0. Then, the docking calculations were carried out using AutoDock Vina 1.1.2. These two software programs are freely available from http://autodock.scripps.edu/downloads. The open-source PyMOL was used to demonstrate the interactions between proteins and ligands.

To further confirm the specificity of the candidate protein for active compounds rather than inactive ones, the binding energy values for docking of the candidate proteins within the test set, which included 17 active amino alcohols and 36 inactive ones (Supplementary Figures 1B,C), were analyzed with receiver operating characteristic (ROC) curve.

### Sequence Amplification and Alignment of *E. granulosus* Glycogen Phosphorylase

The sequence of *Homo sapiens* glycogen phosphorylase (Gp, NP_002854.3) was used for a BLAST search of the cDNA database of *E. granulosus*^7^ with *BLAST/N*^8^ which identified two genes encoding Gp in *E. granulosus*. These genes were amplified with Ex *Taq* DNA polymerase (Takara, Japan) using gene-specific primers. For *E. granulosus* Gp (EgGp) 1, the primers were 5′-ATGTCCTTAGATGAATAT-3′ (forward) and 5′- CTACTTGCTGGAGGTAGC-3′ (reverse). For EgGp2, the primers were 5′-ATGTCTCTCGATAAGCTT-3′ (forward) and 5′-CTACTTGGCGGCGGCAGG-3′ (reverse). The PCR mixture contained primers (1 μM each), a dNTP mixture (200 μM), 1 × PCR buffer and 0.5 units of Ex*Taq* DNA polymerase. The PCR conditions were as follows: denaturation for 5 min at 95°C, 35 cycles of amplification (40 s at 95°C, 30 s at 60°C, 90 s at 72°C), and extension for 10 min at 72°C. The PCR products were separated on 1.2% agarose gels and purified with a Gel Extraction Kit (Qiagen, Germany). The purified PCR fragments were directly cloned into the pMD19-T vector (Takara, Japan) using a Mighty TA-Cloning Kit (Takara) and transformed into *Escherichia coli* DH5a cells (Tiangen, China). A single clone for each construct was selected and sequenced (Sunny Biotechnology, Co., Ltd., Shanghai, China). The sequence analyses and alignments were performed using MEGA 6.0^9^ and Clustal Omega^10^.

### Detection of Gp in *E. granulosus* Protoscoleces and Cysts by Quantitative RT-PCR (qPCR)

*Echinococcus granulosus* G1 PSCs and inner cysts without PSCs from infected sheep were prepared as previously reported (Liu et al., 2018). To create a template for first-strand cDNA synthesis (Takara), total RNA was extracted with an RNeasy Mini Kit (Qiagen). The specific primer sequences for amplification were as follow: EgGp1, 5′-TTCTACGTGATCGCACAGTACA-3′ (forward) and 5′-GGAAAACCAGCTTGAGT-3′ (reverse); EgGp2, 5′-ACGCGAAACGTTCCACAGCTTC-3′ (forward) and 5′-CCTCCATAATCTCATCTGACAG-3′ (reverse); and EF-1α (internal control; Espinola et al., 2014), 5′-TTTGAGAAAGAG GCGGCTGAGATG -3′ (forward), and 5′-TAATAAAGTCAC GATGACCGGGCG-3′ (reverse). The qPCR mixture contained primers (0.4 μM each) and 12.5 μl of SYBR Green Real-time PCR Master Mix (Toyobo, Japan). The cycling protocol was as follows: 95°C for 30 s, followed by 40 cycles of 95°C for 5 s, 60°C for 10 s, and 72°C for 30 s. Melting curves were generated by cooling the products to 65°C and then heating them to 95°C at a rate of 0.1°C/s while simultaneously measuring fluorescence. The qPCR products were separated on 2.0% agarose gels (Supplementary Figure 2A). Quantification of mRNA were normalized with internal reference gene (EF-1α), the difference of mRNA expression was analyzed by *t*-test using SPSS version 17.0, *P* < 0.05 was considered statistically significant.

### Western Blotting and Antibodies

*Echinococcus granulosus* PSCs collected from liver hydatid cysts of newly slaughtered sheep were homogenized with lysis buffer at 4°C and centrifuged at 8000 × *g* for 15 min. Then, 20 μg of the protein-containing supernatant was analyzed by sodium dodecyl sulfate-polyacrylamide gel electrophoresis (SDS-PAGE), and the Polyvinylidene fluoride (PVDF) membrane was blocked for 1 h and incubated with anti-PYGB (Abcam, United Kingdom, ab154969), anti-PYGL (Abcam, ab198268) and anti-PYGM (Abcam, ab231963) primary antibodies (1/1,000) at 4°C overnight. Then, the membrane was incubated with a 1/5,000 dilution of a goat anti-rabbit IgG secondary antibody conjugated with HRP (Abcam, United Kingdom, ab6721) at room temperature for 1 h before washed again. An ECL Western Blotting Substrate Kit (Tanon, China) was used to detect the proteins on the PVDF membrane.

### Immunoprecipitation and Protein Identification

Commercial Gp antibodies (10 μg) were immobilized on 50 μl of Dynabeads Protein A (Invitrogen, United States) according to the manufacturer’s protocol. *E. granulosus* PSC lysates were incubated with antibody/Dynabeads Protein A for 10 min at room temperature. The immunoprecipitates were washed three times in wash buffer and eluted. Protein identification was performed by Shanghai Applied Protein Technology, Co., Ltd., on a Q Exactive mass spectrometer (Thermo Fisher Scientific, United States).

### Immunofluorescence and Confocal Microscopy

Collected *E. granulosus* PSCs were washed with Phosphate-buffered saline (PBS) three times. Then, the samples were fixed in 4% paraformaldehyde at room temperature for 1 h, washed with PBS three times, and permeabilized by 30-min treatment with proteinase K (20 μg/ml, Fermentas, Germany) at 37°C. Then, the samples were washed three times with PBS, treated with 0.1% Triton-X 100 for 15 min, blocked with 3% BSA for 1 h at room temperature, incubated with a 1/50 dilution of Gp antibodies at 4°C overnight and incubated with goat anti-rabbit IgG H&L Alexa Fluor 555 (Abcam, ab150078) for 1 h at room temperature. Before observation by confocal microscopy (Nikon, Japan), 4′,6-diamidino-2-phenylindole (DAPI) solution was added to each sample.

**TABLE 1:** Putative *Echinococcus granulosus* drug targets.

Eg ID	Genome ID	Protein Name	PDB ID	Protein Name	Origins
Eg1	EgrG_000095900.1	Lysine specific histone demethylase 1A	2HKOA	Lysine-specific histone demethylase 1	*Homo sapiens*
Eg2	EgrG_000098400.1	ATP dependent RNA helicase DDXX	2DB3A	ATP-dependent RNA helicase vasa	*Drosophila melanogaster*
Eg3	EgrG_000115600.1	ATP synthase gamma subunit	1H8EG	Bovine mitochondrial F1-ATPase	*Bos Taurus*
Eg4	EgrG_000120200.1	Inosine 5’ monophosphate dehydrogenase 2”	2A7RA	GMP reductase 2	*Homo sapiens*
Eg5	EgrG_000144900.1	Mitogen activated protein kinase 14	1DI9A	P38 KINASE	*Homo sapiens*
Eg6	EgrG_000155600.1	Aldo keto reductase family 1 member B4	1Q5MA	Prostaglandin-E2 9-reductase	*Oryctolagus cuniculus*
			2FVLA	Aldo-keto reductase family 1, member C4	*Homo sapiens*
			3D3FA	YvgN protein	*Bacillus subtilis*
Eg7	EgrG_000156400.1	Aldo keto reductase family 1 member B4	1K8CA	Xylose reductase	*Candida tenuis*
Eg8	EgrG_000184700.1	Histone h3 methyltransferase	1NW3A	Histone methyltransferase DOT1L	*Homo sapiens*
Eg9	EgrG_000190000.1	Ferredoxin	3LB8C	Putidaredoxin	*Pseudomonas putida*
Eg10	EgrG_000210300.1	Lysine specific histone demethylase 1A	2BK3A	Amine oxidase [Falvin-cotaning] B	*Homo Sapiens*
			2VVLA	Monoamine Oxidase N	*Aspergillus Niger*
Eg11	EgrG_000245100.1	Endoplasmic reticulum oxidoreductin 1	1RP4A	Hypothetical 65.0 kDa protein in COX14-COS3 intergenic region precursor	*Saccharomyces cerevisiae*
Eg12	EgrG_000297300.1	BC026374 protein S09 family	1Q83A	Acetylcholinesterase	*Mus musculus*
Eg13	EgrG_000315200.1	Acyl coenzyme A dehydrogenase family	1JQIA	Acyl-CoA dehydrogenase	*Rattus norvegicus*
Eg14	EgrG_000422600.1	Aldo keto reductase family 1 member B4	1KNRA	L-aspartate oxidase	*Escherichia coli*
Eg15	EgrG_000439900.1	Diphthine synthase	2Z6RA	Diphthine synthase	*Pyrococcus horikoshii*
Eg16	EgrG_000445600.1	Long chain fatty acid coenzyme A ligase 5	2D1RA	Luciferin 4-monooxygenase	*Luciola cruciata*
Eg17	EgrG_000457900.1	Casein kinase ii subunit alpha	2OXDA	Casein kinase II subunit alpha	*Zea mays*
Eg18	EgrG_000485300.1	/product = spermidine synthase	2E5WA	Probable spermidine synthase	*Pyrococcus horikoshii*
			2PT9A	Spermidine synthase	*Plasmodium falciparum*
Eg19	EgrG_000501500.1	Glycogen phosphorylase	1KTIA	Glycogen phosphorylase, muscle form	*Oryctolagus cuniculus*
Eg20	EgrG_000523800.1	Aldo keto reductase family 1 member B4	1FRBA	FR-1 PROTEIN	*Mus musculus*
Eg21	EgrG_000599900.1	NADPH:adrenodoxin oxidoreductase	1CJCA	Adrenodoxin oxidoreductase	*Bos taurus*
Eg22	EgrG_000608500.1	Lactate dehydrogenase a	2A94A	L-Lactate dehydrogenase	*Plasmodium falciparum*
Eg23	EgrG_000618700.1	Glutamate receptor ionotropic kainate 2	3ILUB	Glutamate receptor 2	*Rattus norvegicus*
Eg24	EgrG_000644000.1	Glutamate synthase	1EA0A	Glutamate synthase [NADPH] large chain	*Azospirillum Brasilense*
Eg25	EgrG_000688200.1	H2A histone family member Y	3IIFA	Core histone macro-H2A.1, Isoform 1	*Homo sapiens*
Eg26	EgrG_000708200.1	Brefeldin A inhibited guanine	1S9DE	ADP-Ribosylation Factor 1	*Bos taurus*
Eg27	EgrG_000717700.1	Chaperonin containing TCP1 subunit 5 epsilon	1Q3SA	Thermosome alpha subunit	Thermococcus sp.
Eg28	EgrG_000720500.1	ATP synthase subunit alpha mitochondrial	1H8EA	Bovine mitochondrial F1-ATPase	*Bos taurus*
Eg29	EgrG_000732400.1	Acetylcholinesterase	1E3QA	Acetylcholinesterase	*Torpedo californica*
Eg30	EgrG_000752000.1	ATP synthase subunit beta mitochondrial	1H8ED	Bovine mitochondrial F1-ATPase	*Bos taurus*
			1H8EH	Bovine mitochondrial F1-ATPase	*Bos taurus*
Eg31	EgrG_000787800.1	Aldo keto reductase family 1 member B4	1HQTA	Aldehyde reductase	*Sus scrofa*
			2PDIA	Aldose reductase	*Homo sapiens*
Eg32	EgrG_000792800.1	3-oxoacyl-(acyl-carrier-protein) reductase	1AE1A	Tropinone reductase-1	*Datura stramonium*
Eg33	EgrG_000820900.1	Methionyl aminopeptidase 2	1R58A	Methionine aminopeptidase 2	*Homo sapiens*
Eg34	EgrG_000831600.1	Histone deacetylase 7	2VQQA	Histone deacetylase 4	*Homo sapiens*
Eg35	EgrG_000832900.1	Subfamily M12B unassigned peptidase	1DTHA	Atrolysin C	*Crotalus atrox*
Eg36	EgrG_000870200.1	Dihydrolipoamide dehydrogenase	2F5ZA	Dihydrolipoyl dehydrogenase	*Homo sapiens*
Eg37	EgrG_000924600.1	Transmembrane protease serine 3	2ZGHA	Granzyme M	*Homo sapiens*
Eg38	EgrG_000939100.1	Matrix metallopeptidase 7 M10 family	1G4KA	Stromelysin-1	*Homo sapiens*
			1XUCA	Collagenase 3	*Homo sapiens*
Eg39	EgrG_000954200.1	Ribosomal RNA processing protein 8	2ZFUA	Cerebral protein 1	*Homo sapiens*
Eg40	EgrG_001032250.1	Aminotransferase class III	1WKGA	Acetylornithine/acetyl-lysine aminotransferase	*Thermus thermophilus*
Eg41	EgrG_001035900.1	Protein arginine N methyltransferase 8	2FYTA	Protein arginine N-methyltransferase 3	*Homo sapiens*
Eg42	EgrG_001043100.1	Phosphoglycerate kinase 1	2PAAA	Phosphoglycerate kinase, testis specific	*Mus musculus*
Eg43	EgrG_001126400.1	FAD linked sulfhydryl oxidase ALR	1JR8A	Erv2 PROTEIN, mitochondrial	*Saccharomyces cerevisiae*
			1OQCA	Augmenter of liver regeneration	*Rattus norvegicus*
Eg44	EgrG_001133400.1	Protein l isoaspartate o methyltransferase	1JG4A	Protein-L-isoaspartate O-methyltransferase	*Pyrococcus furiosus*
Eg45	EgrG_001153000.1	Mitochondrial F1F0 ATP synthase subunit epsilon	1H8EI	Bovine mitochondrial F1-ATPase	*Bos Taurus*
Eg46	EgrG_001170100.1	Histone lysine methyltransferase setb	3K5KA	Histone-lysine N-methyltransferase, H3 lysine-9 specific 3	*Homo sapiens*
Eg47	EgrG_001171200.1	Biogenic amine 5HT receptor	2RH1A	Beta-2-adrenergic receptor/T4-lysozyme chimera	*Homo sapiens*, Enterobacteria phage T4
Eg48	EgrG_001176600.1	NAD dependent deacetylase sirtuin 3	3D4BA	NAD-dependent deacetylase	*Thermotoga maritima*
Eg49	EgrG_001177600.1	ADP ribosylation factor 4	1S9DA	ADP-Ribosylation Factor 1	*Bos taurus*

### Activity of Compounds on Gp in *E. granulosus* PSC Homogenate

The activity of Gp was tested in a glycogen degradation assay by coupling the production of glucose-1-phosphate to the reduction of Nicotinamide adenine dinucleotide phosphate (NADP) using phosphoglucomutase and glucose-6-phosphate (G6P) dehydrogenase. The assay medium for the measurement of Gp contained 50 mM Tris-HCl buffer, 1.4 mM dithiothreitol (DTT), 1 mM EDTA, 5 mM MgCl_2_, 0.6 mM NADP, 5 μM glucose-1,6-bisphosphate, 0.5 U of phosphoglucomutase, 0.35 U of G6P dehydrogenase, 2 mg/ml glycogen and 1 mM 5′-adenosine monophosphate (5′-AMP). After co-incubation with 0–200 μg/ml of the test compounds at 25°C for 2 min, the activities of recombinant rabbit Gp and *E. granulosus* PSC homogenate were determined by observing the absorbance at 340 nm for 30 min. The endogenous NADP dehydrogenase activity was corrected via analysis of a blank without added enzymes, glycogen and AMP. One unit of Gp was defined as the amount required to form 1 μmol of glucose-1-phosphate/min at 25°C (Arrese et al., 1995). For both enzymes, the assay was performed in 96-well plates and repeated three times (*n* = 3). The raw data were processed in Microsoft Excel. The inhibition rates of each test compound and the positive control were obtained using the formula

$$I\;\left(\%\right)=\left[\left(E_{0}-E_{i}\right)/E_{0}\right]\times 100,$$

where *I* (%) is the inhibition rate, *E*_i_ is the enzyme activity in the presence of the compound, and *E*_0_ is the enzyme activity in the absence of the compound.

The concentration of each sample that inhibited 50% of the enzyme activity (IC_50_) was calculated by the probability unit method with SPSS version 17.0.

## Results

### Semiautomatic *in silico* Workflows to Identify Candidate *E. granulosus* Drug Targets

**FIGURE 1:**
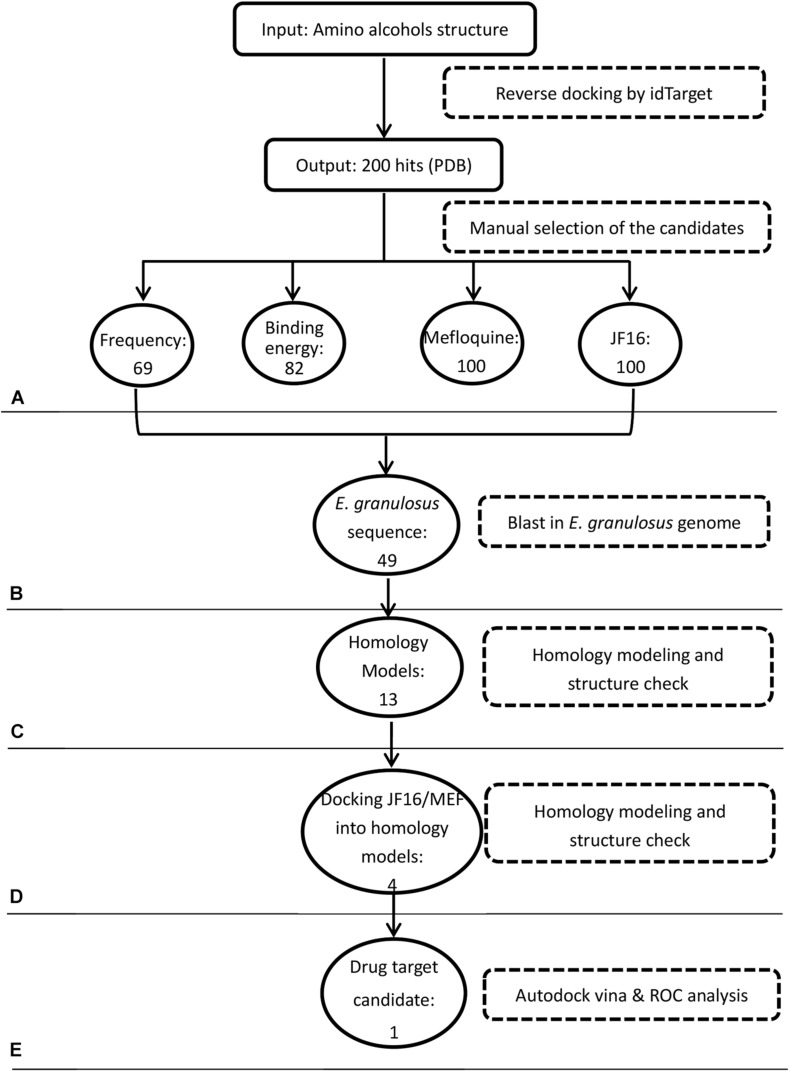
Workflow of target identification for amino alcohols. **(A)** Protein target prediction with idTarget and selection of candidate targets. **(B)** BLAST search against the *Echinococcus granulosus* genome. **(C)** Homology modeling. **(D)** Molecular docking with active amino alcohols. **(E)** Target validation by receiver operating characteristic (ROC) curve analysis.

In the first step of the *in silico* workflow (Figure 1), the 11 most active amino alcohols (Supplementary Figure 1A) identified in the first screening using *in vitro-*cultured *E. granulosus* were inputted into the idTarget web server. The inverse docking system outputted and ranked the target proteins of these compounds (Supplementary Table S1). To select the common targets of the amino alcohols, we identified 64, 82, 100, and 100 candidates respectively, according to the frequency, binding energy, top 100 proteins of JF16 and top 100 proteins of MEF. The amino acid sequences of the above proteins were pooled and blasted against the entire *E. granulosus* genome database to identify the orthologues. A total of 49 *E. granulosus* proteins were identified that corresponded to 57 PDB sequences (Table 1). Then, homology models were generated based on the amino acid sequences of these 49 *E. granulosus* proteins with MODELLER 9.13. Moreover, the structures of these models were assessed with SAVES, and only those with suitable PROCHECK (> 95%), ERRAT (> 90) and VERIFY 3D (> 80%) values were subjected to molecular docking (Supplementary Table S2). Then, the resulting 13 *E. granulosus* homology models were docked with the two most effective amino alcohols, JF16 and MEF. The lowest calculated binding energy and docking poses are listed in Supplementary Table S2. According to these data, we identified four *E. granulosus* proteins (parasite proteins) as potential drug targets. The test set (Supplementary Figure S1) was prepared to confirm the specificity of the candidate proteins for active compounds rather than inactive ones. Ultimately, only one *E. granulosus* protein was selected as a potential drug target of amino alcohols on the basis of an area under the curve (AUC) values higher than > 0.7 in the ROC curve analysis (Supplementary Table S2).

### Sequence Analyses of EgGps and Predicted Interactions of MEF and JF16 With EgGps

**FIGURE 2:**
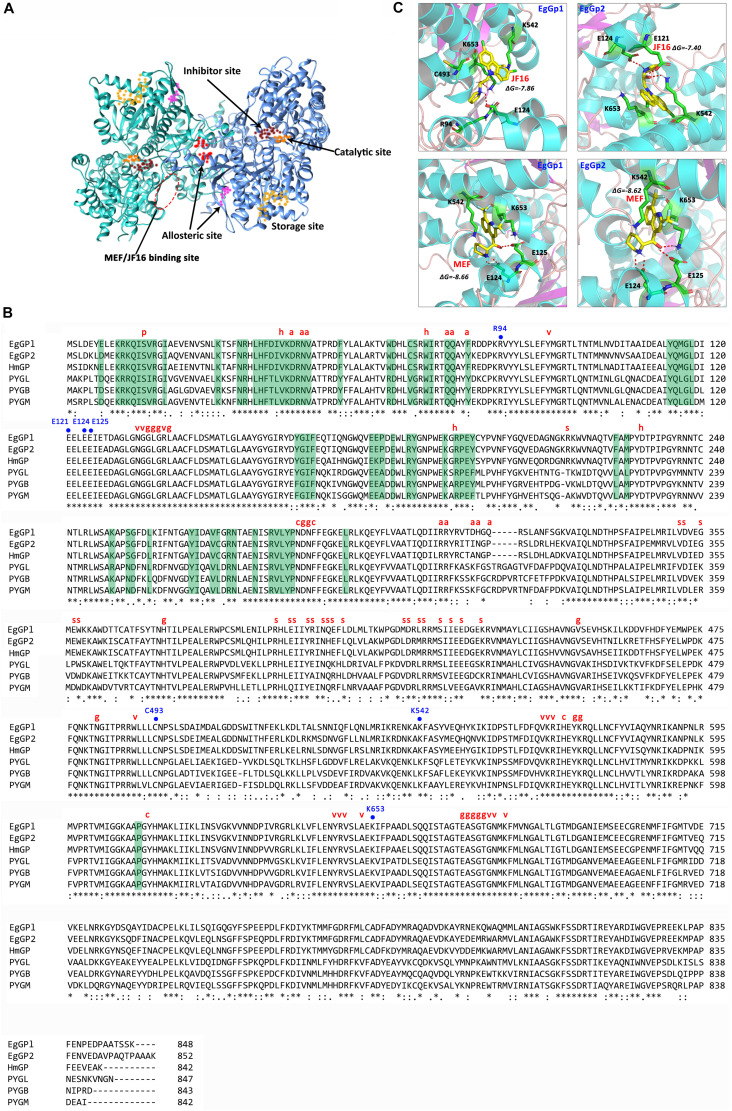
Predicted structure and sequence alignments of EgGps. **(A)** Modeled structure of EgGp1. **(B)** Sequence alignments of EgGps and corresponding amino sequences from other species. “^∗^” symbols denote the positions of amino acids that have a single fully conserved amino acid residue; “:” symbols denote conservation between groups of amino acids with strongly similar properties; “.” symbols denote conservation between groups of amino acids with weakly similar properties; and “–” symbols denote gaps inserted to maximize sequence alignment. Amino acid side chains that participate in subunit contact or ligand binding are labeled as follows: dimer contact (green background); p, Ser phosphorylation site; h, G6P binding site; a, nucleotide activator site (AMP binding site); v, PLP- binding residue; g, active site residues (glucose binding); s, glycogen storage site; c, purine inhibitor (caffeine) site; EgGp1, *Echinococcus granulosus* glycogen phosphorylase 1 (MN562583); EgGp2, *E. granulosus* glycogen phosphorylase 2 (MN562584); HmGP, *Hymenolepis microstoma* glycogen phosphorylase (NP_002854.3); PYGL, *Homo sapiens* glycogen phosphorylase, liver form (NP_002854.3); PYGB, *Homo sapiens* glycogen phosphorylase, brain form (NP_002853.2); and PYGM, *Homo sapiens* glycogen phosphorylase, muscle form (NP_005600.1). **(C)** Analysis of compound docking to the target proteins; the red dashes represent H-bonds.

By performing a BLAST search against the *E. granulosus* genome, we found two *E. granulosus* genes encoding Gps. The cDNA of these two genes was amplified and sequenced. We named these two genes EgGp1 (GenBank: MN562583) and EgGp2 (GenBank: MN562584). The similarity of the deduced amino acids of these two sequences was 85.81%, close to the similarity (86.24%) of three human Gps (glycogen phosphorylase, liver form, PYGL; glycogen phosphorylase, brain form, PYGB; glycogen Phosphorylase, muscle form, PYGM). Moreover, the identities of EgGp1 and EgGp2 with *Hymenolepis microstoma* Gp were 83.49 and 87.91%, respectively, higher than their identities with the human proteins, which ranged from 62.33 to 65.35%; these findings indicate the lineage specificity of Gp proteins during evolution. The characteristics of the amino acids in the functional sites are shown in Figures 2A,B. The S14 site, the catalytic site (g), the G6P binding site (h), pyridoxal phosphate (PLP)-binding residues (v), and the purine inhibitor site (c) were highly conserved in both tapeworm and human phosphorylases, suggesting their crucial roles in the functions of these enzymes. However, fewer conserved residues were found in the nucleotide activator site (a), the glycogen storage site (s), and sites (d) of inter-subunit contact in the dimer. The nucleotide activator site was found to form contacts with AMP, which is required for catalytic activity. At these sites, the polar tyrosine (Y73) in humans was replaced with non-polar phenylalanine (F73) in *E. granulosus* and *H. microstoma.* In addition, K317, F318 and S/C320 in humans Gps were also replaced with different amino acids in the tapeworm Gps. The glycogen storage site was highly conserved in the tapeworm, but differences in the residues were observed between tapeworm Gps (L400) and human Gps (R or H at 414). The dimer contact residues, which are required for allosteric effects, showed diversity in humans and parasites. These less conserved residues support EgGps as potential specific drug targets for the treatment of echinococcosis.

The amino acid sequences were deduced from the cDNA sequences of the EgGps, and used to build 3D models. The docking poses were obtained by docking JF16 and MEF into the two EgGps (Figure 2C). Both of JF16 and MEF docked into the same pocket of EgGp1 and EgGp2. At these positions, these compounds formed contacts with K542 and K653 via pi-stacking interactions. In addition, the interactions were also supported by H-bonds and charges at these positions. The residues in the MEF/JF16 binding site were highly conserved in both parasites and humans.

### Expression, Localization, and Protein–Protein Interaction Analyses of EgGps

**FIGURE 3:**
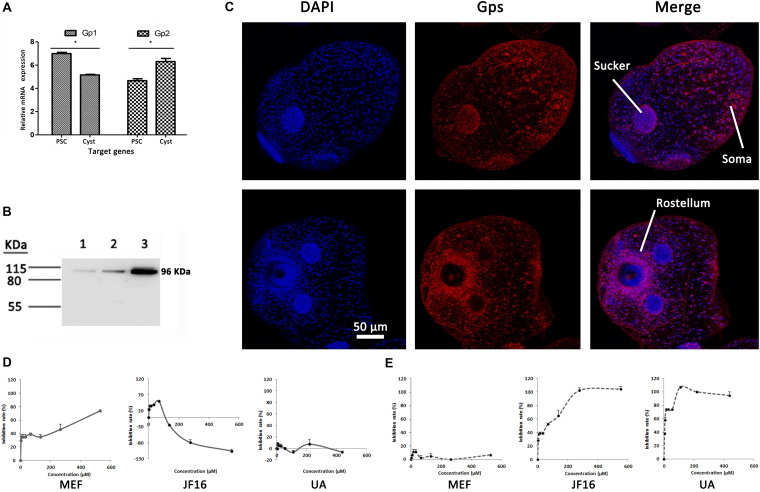
Relative mRNA expression levels (ΔCt values) and localization of EgGps, and effects of compounds on EgGps. **(A)** Relative mRNA expression levels of the target genes, ΔCt = (Ct of target genes) – (Ct of EF-1α). PSCs, protoscoleces. *P < 0.01. **(B)** Gps in *Echinococcus granulosus* PSC lysates (20 μg of total protein); lane 1: anti-PYGM, lane 2: anti-PYGL, lane 3: anti-PYGB. **(C)** Localization of Gps in *E. granulosus* PSCs (confocal microscopy, anti-PYGB antibody). The anti-PYGM antibody failed to label proteins and the anti-PYGL yielded results similar to those yielded by the anti-PYGB antibody. **(D)** The effects of mefloquine (MEF), JF16, and ursolic acid (UA) on EgGps *in vitro*. **(E)** The effects of MEF, JF16, and UA on Rabbit muscle Gps *in vitro*.

Specific primers were designed to analyze the expression levels of EgGp1 and EgGp2 mRNA in *E. granulosus* PSCs and cysts (Supplementary Figure S2). The expression levels of EgGp1 in PSC samples were higher than those in cysts, while the levels of EgGp2 showed the opposite trend (*P* < 0.05). That is, EgGp1 was abundant in *E. granulosus* PSCs, while EgGp2 was abundant in the *E. granulosus* cysts (*P* < 0.05; Figure 3A). Since there are no specific antibodies for EgGp1 and EgGp2, commercial Gp antibodies (anti-PYGM, anti-PYGL, and anti-PYGB) were used in this experiment. A single band at 96 kD was recognized by all three antibodies in the total protein of *E. granulosus* PSCs, indicating the specificity of these antibodies for EgGps (Figure 3B).

These antibodies were also used to pull down the Gps and interacting proteins in parasite lysates. The anti-PYGB and anti-PYGM antibodies were able to pull down EgGps in the eluent, while the anti-PYGL antibody failed to label EgGps. However, the mass spectra did not distinguish the two isoforms of EgGps. In addition, proteins that potentially interacted with the EgGps were identified in the eluent (Supplementary Table S2). These proteins included hydrocephalus inducing homology and three uncharacterized proteins, indicating that EgGps may be involved in some *E. granulosus*-specific functions.

The EgGps in PSCs and in cysts that developed from PSCs *in vitro* were localized with an anti-PYGB antibody. As shown in Figure 3, EgGps were mostly found in the suckers and somas of *E. granulosus* PSCs, which are responsible for the movement of the parasite with the consumption of energy. When cysts develop from PSCs, the movement of the parasites can no longer be observed. Hence, the EgGps were not concentrated at the same sites in cysts as in PSCs. However, interestingly, these enzymes were found near the rostellum.

### Effects of Amino Alcohols on Gps From Parasites and Hosts *in vitro*

To confirm the interactions of MEF and JF16 with EgGps, the effects of these compounds on the activity of EgGps in *E. granulosus* PSC homogenate were evaluated *in vitro* (Figures 3D,E and Table 2). MEF inhibited the activity of EgGps in a concentration-dependent manner, but the IC_50_ value of MEF for EgGp was nearly thirty times that of the parasites *in vitro*, indicating that there are other modes of action for MEF. The performance of JF16 was quite different from that of MEF. Specifically, at low concentrations (4.31-69.00 μM), JF16 inhibited up to 50% of the activity of Gps in *E. granulosus* PSCs. Surprisingly, when the concentration increased, the enzyme activity in *E. granulosus* was promoted. In addition, UA had no impact on the activity of Gps in the *E. granulosus* PSC homogenate. UA, a reported Gp inhibitor, inhibited the activity of Gps from rabbit muscle at very low concentrations. The effects of JF16 on the same rabbit muscle Gps were similar to UA, while MEF showed no effects under the same conditions.

**TABLE 2:** IC_50_ (μM/mL) of the compounds on activity of parasites and glycogen phosphorylase in parasites.

Compounds	IC_50_/LC_50_ (μM)			
	*Echinococcus* spp.		Gp	
	PSCs	GC	Rabbit muscle	*E. granulosus* PSC
MEF	5.77^a^	4.47^a^	> 437.92	128.29
JF16	18.42^a^	5.04^a^	31.57	ND^b^
UA	> 87.59^c^	12.39^c^	5.474	> 437.92

*Results obtained are expressed as mean of three replicate. PSC, protocoleces; GC, germinal cells; Gp, glycogen phosphorylase; ND, not determined. ^*a*^(Liu et al., 2018). ^*b*^IC_50_ value can’t be calculated from inhibition rate data. ^*c*^Calculated from our published paper (Yin et al., 2018).*

## Discussion

Echinococcosis is a neglected disease that has been of very limited interest to the pharmaceutical industry in terms of drug development (Siles-Lucas et al., 2018). None of the candidate alternative drugs or compounds tested on *Echinococcus* spp. was first designed for the treatment of CE or AE. In addition, poor understanding of drug modes of action limits target-based drug screening and design in related studies. Recently, we focused on a series of amino alcohols (Liu et al., 2018; Liu et al., 2020) because MEF, which is used for the treatment of malaria and schistosomiasis, has recently been reported to be effective against *Echinococcus* spp. (Kuster et al., 2011; Liu et al., 2015; Rufener et al., 2018). The mechanism of MEF has been found to be related to haematin (Xiao et al., 2014; Herraiz et al., 2019), which is not present in the parasitic environment of *Echinococcus* spp. Hence, it is assumed that MEF must inhibit these parasites in other ways, which is what we sought to elucidate in this study.

In recent decades, protein-based virtual screening has been widely used (Bissantz et al., 2000; Bissantz et al., 2003) and has been facilitated by increasing amounts of protein structure information in the PDB (Berman et al., 2002; Burley et al., 2019). The current study is the first to predict the drug targets of amino alcohols in *Echinococcus* spp. by inverse docking using idTarget, a web server that aids in identification of the protein targets of small chemical molecules with robust scoring functions and a divide-and-conquer docking approach (Wang et al., 2012). The scoring system uses AutoDock4RRP, AutoDock4RAP, and AutoDock4RGG in AutoDock4, which create a regular map inside the proteins. Then, a ligand or fragment is placed as the probe on the different poses of the map, and finally, the interaction energy is calculated by evaluating the electric charge using RESP or AM1-BBC model. The poses can also be identified at the same time (Wang et al., 2012). This system has been used to explore the drug targets of many compounds (Bhattacharjee et al., 2012; Chen and Ren, 2014; Smelcerovic et al., 2014). However, among the nearly 20,000 proteins in the PDB, only hundreds belong to parasites (most of from are from protozoans); thus, it was not surprising that all the targets predicted by idTarget were from other species. Hence, we built homology models based on the sequence identity between these idTarget predicted proteins and their homologues in *E. granulosus*. After docking amino alcohols to these *E. granulosus* homology models, Gp was identified as the candidate drug target for these compounds.

Glycogen is a multi-branched polysaccharide of glucose that serves as the main energy storage molecule in the body. Gp, the rate-limiting enzyme in the breakdown of glycogen, cleaves the non-reducing ends of glycogen to produce monomers of glucose-1-phosphate, which is ultimately converted to G6P by phosphoglucomutase (Adeva-Andany et al., 2016). G6P is then used as a substrate for glycolysis or, in gluconeogenic tissues, enters the endoplasmic and sarcoplasmic reticulum through a G6P transporter and is converted to glycogen by glucose-6-phosphatase (Prats et al., 2018). In humans, there are three isoforms of Gp: liver, muscle and brain isoforms. Notably, disorders of glycogen metabolism are related to many diseases (Ritterson Lew et al., 2015; Goyard et al., 2016). The glycogen metabolism pathway is also involved in cancer development, and the enzyme Gp has been targeted by inhibitors as a tumor promoter in preclinical studies (Ritterson Lew et al., 2015). In addition, the important role of Gp in the parasites life cycle (Tandon et al., 2003; Sugi et al., 2017) makes this enzyme a potential drug target (Tandon et al., 2003). However, research on Gp in *Echinococcus* spp. is limited. We identified two Gp isoforms in the *E. granulosus* genome by BLAST search and the different expression levels of these Gps in PSCs and cysts indicate diversity in their function or regulation. The strong fluorescence signals in PSC suckers and somas indicated high level of the Gps in these regions, consistent with the high energy levels required for the movement of these parasites in their PSC form. Movement is lost when the PSCs developed into cysts; accordingly, the fluorescence signals at the suckers and somas were weakened in cysts. In addition, high levels of Gp were observed near the rostellum, which was crowded with cells, suggesting that cellular activity such as cell division may require energy from glycogen degradation that is contributed by Gp.

According to the structural characteristics of Gp, this protein is a typical allosteric enzyme with at least six different binding sites, and these sites have also been identified as the targets of many compounds (Deng et al., 2005). Sequence alignment revealed that these binding sites were also present in EgGps and showed residue diversity between humans and parasites, a characteristic that can be used for target-based drug design and screening. The binding sites for MEF and JF16 on EgGps were predicted to be pockets formed by E124, K542, and K653. These are novel binding sites indicating that amino alcohols exhibit different mechanisms in inhibiting the activity of Gps. It is noticed that these residues were conserved in both of the parasite and the host, indicating that there are more amino acids involved in the interaction of these compounds with EgGps. However, this is the predicted docking pocket, more experiments, such as the mutation of crucial residues will reveal the accurate residues responsible for the activity of EgGps and for protein-ligand interaction. Based on these data, together with molecular dynamics simulations which can evaluate the molecular flexibility of ligands and receptors, more accurate docking and virtual screening methods (Tao et al., 2019; Wang et al., 2020), the promising hits targets EgGps will be found and benefit the development of anti-echinococcal compounds. The interactions of these compounds with Gps were proven *in vitro* by determining the activity of Gps in PSC homogenates. Unlike UA, MEF did not inhibit rabbit muscle Gps *in vitro*, which supports our speculation that MEF can selectively inhibit EgGps rather than the host Gps. Moreover, we found that several proteins may engage in protein-protein interactions with EgGp1, the functions of which are unexplored. As the promising drug targets for *Echinococcus* spp., this information on EgGps will drive further echinococcal drug research in the future.

## Conclusion

In our study, we predicted the drug targets of amino alcohols using inverse docking, a new method for studying the mechanisms of active compounds against *Echinococcus* spp. The novel findings of this study, including the results from *in silico* and related molecular biological experiments, underscore the importance of Gps as possible drug candidates. Future studies in this line should include *in vivo* animal studies.

## Data Availability Statement

The datasets generated in this study can be found in online repositories. The names of the repository/repositories and accession number(s) can be found in the article/Supplementary Material.

## Author Contributions

HZ, WH, and CL developed the concept of the work. CL carried out the experiment. CL and JY discussed and analyzed the results. CL, JY, and HZ wrote the manuscript. All authors contributed to the article and approved the submitted version.

## Conflict of Interest

The authors declare that the research was conducted in the absence of any commercial or financial relationships that could be construed as a potential conflict of interest.

## Footnotes

1 http://www.rcsb.org/pdb/home/home.do

2 http://www.sanger.ac.uk/resources/downloads/helminths/echinococcus-granulosus.html

3 https://www.ncbi.nlm.nih.gov/guide/data-software/

4 http://salilab.org/modeller/

5 https://swissmodel.expasy.org/

6 http://services.mbi.ucla.edu/SAVES/

7 http://www.sanger.ac.uk

8 https://blast.ncbi.nlm.nih.gov/Blast.cgi

9 http://www.megasoftware.net

10 http://www.ebi.ac.uk/Tools/msa/clustalo/
