# Supplementary material for: Glycogen Phosphorylase: A Drug Target of Amino Alcohols in Echinococcus granulosus, Predicted by a Computer-Aided Method
Source: Front Microbiol. 2020 Nov 23;11:557039. doi: 10.3389/fmicb.2020.557039 (PMC7719768; doi:10.3389/fmicb.2020.557039)
Supplement: Supplementary file 3 [file Data_Sheet_1.PDF]

Supplementary Material

1 Supplementary Figures and Tables

1.1 Supplementary Figures

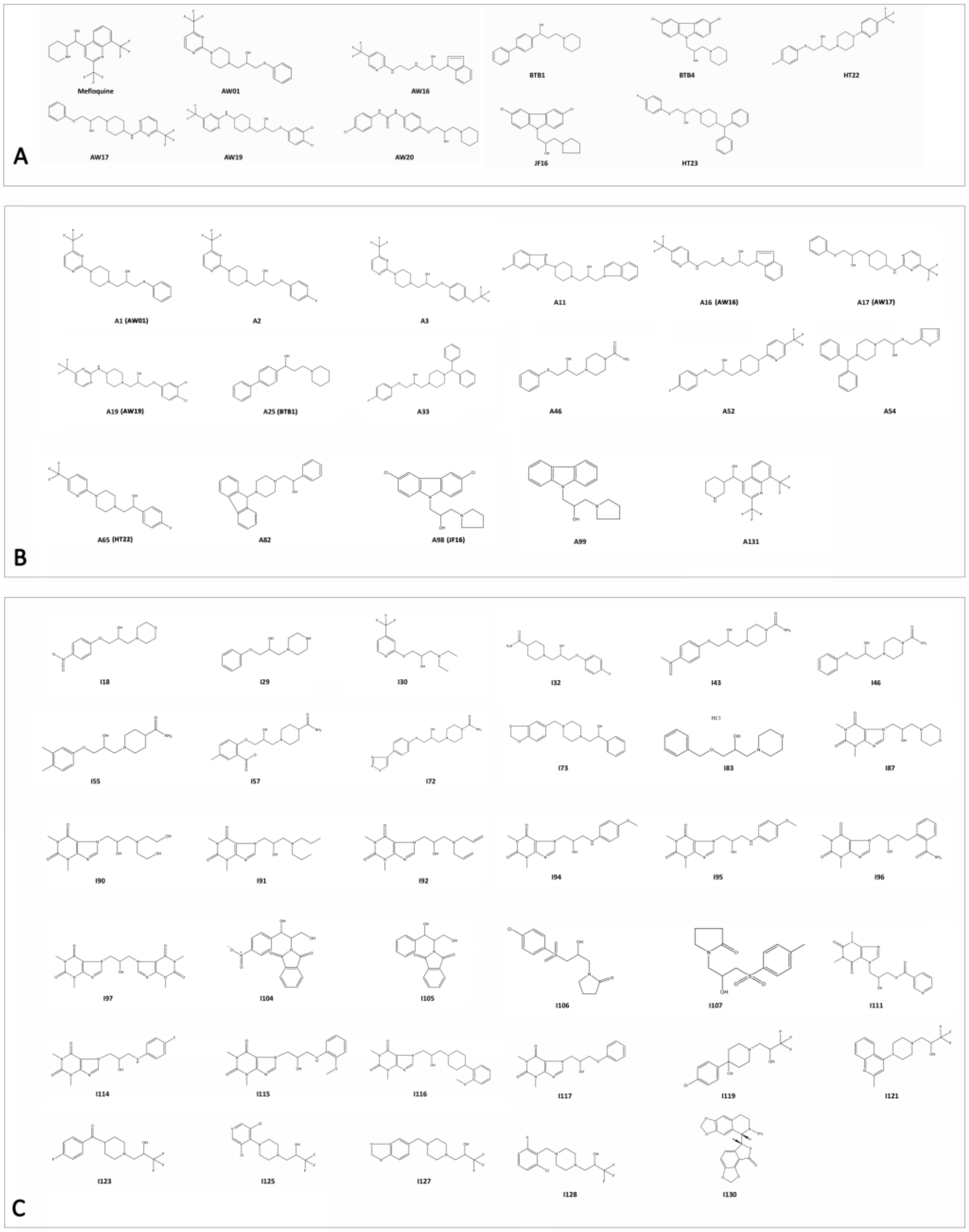

Supplementary Figure 1. Structures of the input (A) and test set (B, active; C, inactive)

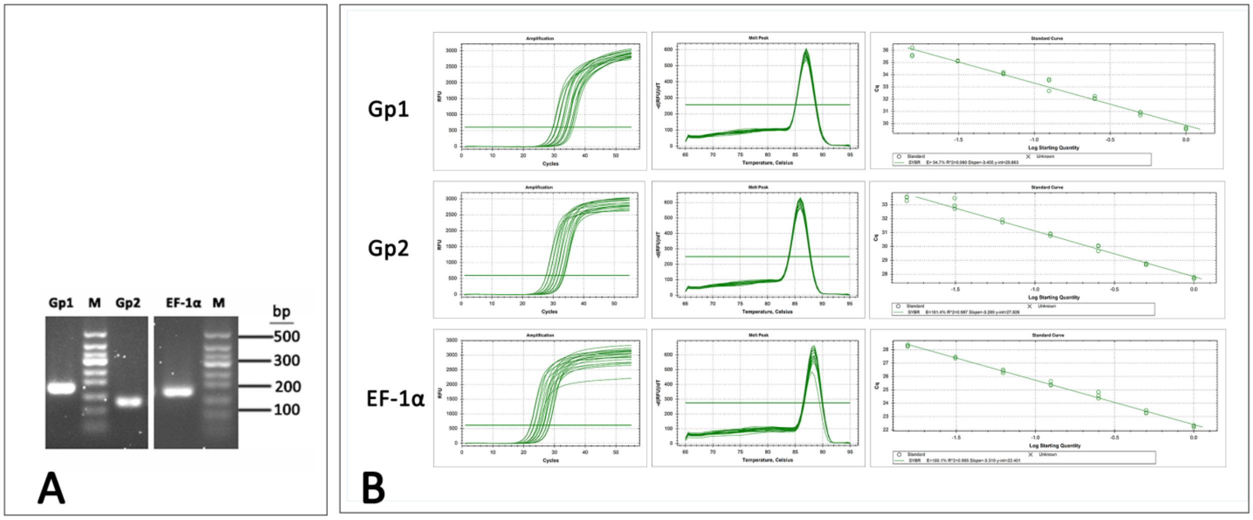

Supplementary Figure 2. Validation of qPCR results. A. Validation of qPCR products in a 2.0% agarose gel (Gp1, 193 bp; Gp2, 125 bp; EF-1α, 174 bp). B. The melting curve and standard curve.
